# Quality of Recovery (QoR)-15 Scale: From Statistical Significance to Clinical Relevance and Beyond

**DOI:** 10.4274/TJAR.2026.252497

**Published:** 2026-06-26

**Authors:** Pranjali Kurhekar, Srinidhi Narayanan, Neeta Parlikar, Buddhan Rajarathinam

**Affiliations:** 1 Institution-Sree Balaji Medical College and Hospital, Department of Anaesthesiology, Critical Care and Pain Medicine, Chennai, India; 2 Kauvery Hospital, Clinic of Critical Care, Chennai, India

**Keywords:** Analgesia, epidural morphine, perioperative care, recovery scale, regional anaesthesia

Dear Editor,

We read with great interest the article titled “Continuous Low-dose Epidural Morphine and Ketamine Analgesia Improves Quality of Recovery (Qor) after Major Lumbar Spine Surgery: A Randomised Controlled Trial” by Karri et al.^1^ We appreciate that the authors compared the effects of continuous epidural opioid (morphine) plus ketamine (Group A) and opioid plus ketamine administered via intravenous (IV) patient-controlled analgesia (Group B) on QoR-15 following major spine surgery in 40 American Society of Anesthesiologists I or II patients scheduled for transforaminal thoracolumbar/lumbar spine instrumentation under general anaesthesia in their randomized clinical trial. Total QoR-15 scores in Group A were found to be significantly better than that of Group B at 24 hours (134.8±6.65 and 128.9±6.12, respectively *P*=0.006) and at 48 hours (136.7±6.02 vs. 132.10±6.8, *P*=0.029) with significantly lower pain scores in group A at rest and during movement. All other secondary outcomes were comparable between the groups. There might be few limitations of of this QoR 15 scale of well proven validity in assessing all dimensions of postoperative recovery^2 ^which could affect the interpretation of the results of Karri et al.^1^

First, the authors concluded that Group A was statistically superior to Group B; the difference in QoR-15 scores was four points at the 48^th^ hour. This can be considered clinically negligible, as a minimal clinically important difference of 6 is needed on the QoR-15 scale to show a meaningful effect of any perioperative intervention.^3^ Due to the nature of the interventions for lumbar laminectomies, significant pain and functional disability are anticipated, with a greater likelihood of changes in QoR scores.^4^ Therefore, measuring preoperative QoR-15 scores could help to determine the clinical significance of postoperative scores.

Second, physical independence is a subdomain of QoR-15 which assesses the ability to do daily activities and work independently. This difference in Karri et al.'s^1^ study is 1.5 points with statistical significance which is clinically meaningless not only by numbers but also taking into account that IV analgesia group received mean morphine dose of 18.25±11.36 mg at the rate of 0.09 mg kg^-1^ hr^-1^ (5.76 mg kg^-1^ hr^-1^ for mean weight of 64 kg with same dose of ketamine in 24 hours), which could potentially affect cognitive and motor functions.^5^

Third, emotional support is another subdomain of QoR 15, which measures feelings of general well-being, comfort, and anxiety or depression. The difference in QoR -15 score of this subdomain in Karri et al.^1^ study was 12 points at 24 hours and one point at 48 hours. At both time points, this difference was statistically significant with the same *P *value of 0.027 (Table 3 of Karri et al.^1^). This identical *P* value in the same sample size could indicate a typographical error or a statistical flaw. Similarly, emotional support scores were low in group B, which received continuous infusion of ketamine and morphine in a 1:1 ratio. Ketamine can cause functional disorganization by its depressive effects on the thalamocortical system and its stimulatory effects on the limbic system. This may also affect the patient’s emotional response and pain scores.^6^ Lastly, one of the subdomains has to be mentioned as psychological support rather than physical support.^1,2^
